# Awareness, Knowledge, and Practices on Glaucoma Among Adults in Rural North Tripura: A Community-Based Cross-Sectional Study

**DOI:** 10.7759/cureus.96807

**Published:** 2025-11-13

**Authors:** Shurekha Debbarma, Aswath R Deepa, Anish Ancil, Ameena A Jaleel, Debashrita Baidya

**Affiliations:** 1 Department of Ophthalmology, Agartala Government Medical College and Govind Ballabh Pant Hospital, Agartala, IND; 2 Department of Internal Medicine, Agartala Government Medical College and Govind Ballabh Pant Hospital, Agartala, IND; 3 Department of Pharmacology, Agartala Government Medical College and Govind Ballabh Pant Hospital, Agartala, IND; 4 Department of Medicine, Travancore Medical College and Hospital, Kollam, IND

**Keywords:** awareness, blindness prevention, community health, eye health, north tripura, rural india, s: glaucoma

## Abstract

Background: Glaucoma is a leading cause of irreversible blindness, yet awareness and early detection remain poor in rural India. Data from North-Eastern India, particularly Tripura, is scarce.

Objective: This study aims to assess awareness, knowledge, and practices (AKP) regarding glaucoma and identify predictors of awareness among adults in rural North Tripura.

Methods: A community-based cross-sectional survey (April-June 2025) was conducted in four randomly selected villages under the District Residency Programme. Multistage cluster random sampling targeted adults aged ≥30 years. A pretested questionnaire (Bengali/Kokborok) captured demographics, awareness, knowledge, practices, and information sources. Descriptive statistics and binary logistic regression identified determinants of awareness.

Results: Of the 420 eligible adults, 368 completed the survey (response rate: 87.6%). A total of 72/368 (19.6%) had ever heard of glaucoma. Among the aware, 25/72 (34.7%) achieved a good awareness score, and 9/72 (12.5%) had good knowledge. Across the full sample, 39/368 (10.6%) reported an eye examination in the past year; among the aware, 17/72 (23.6%) had an exam. Information sources among the aware included health workers, 29/72 (40.3%); eye camps, 20/72 (27.8%); mass media, 13/72 (18.1%); and family/friends, 10/72 (13.8%). Higher education (adjusted odds ratio (AOR): 3.05; 95% CI: 1.87-4.98; p < 0.001) and prior ophthalmic consultation (AOR: 4.92; 95% CI: 2.68-9.02; p < 0.001) independently predicted awareness.

Conclusions: Less than one-fifth of rural adults in North Tripura were aware of glaucoma, and misconceptions were common. Educational status and prior eye-care contact strongly influenced awareness. Community-based education led by trained health workers and integration of glaucoma messaging into routine eye camps may enhance early detection and reduce avoidable blindness.

## Introduction

Glaucoma is a chronic, progressive optic neuropathy and an important cause of irreversible vision loss worldwide. It remains a major contributor to global blindness and continues to account for a large share of permanent bilateral visual impairment, despite public health efforts to reduce avoidable blindness [1]. The global burden of glaucoma is expected to rise as populations age; projections suggest a continuing increase in the number of people affected through 2040, particularly in low- and middle-income regions [2]. In South Asia, pooled population-based estimates indicate that glaucoma affects about 2% of adults, with risk increasing sharply with age and appearing higher in men. Primary open-angle glaucoma is generally more common than primary angle closure glaucoma in older adults [3].

Glaucoma is often asymptomatic in its early stages, and once vision is lost, it cannot be restored. For this reason, it is often referred to as the “silent thief of vision,” reflecting that people may not notice visual field loss until damage is advanced [4-8]. Because of this silent course, prevention depends on two elements: people must know that glaucoma exists and can cause permanent blindness even without early symptoms, and they must have access to examination and follow-up.

Multiple studies from India show that both elements are weak. Population surveys in rural, peri-urban, and urban settings consistently report that only a small proportion of adults have even heard of glaucoma, often below 10-15% [4-8]. Misconceptions are common, including confusion between glaucoma and cataract, and the belief that glaucoma always causes warning symptoms early. These studies also show that awareness is socially patterned. Adults with more formal education or with prior contact with an eye care provider are more likely to know that glaucoma can lead to irreversible vision loss and may run in families, while those without such contact often remain unaware until vision is already affected [4-8].

The North Eastern region of India, including Tripura, faces additional structural barriers. The population is largely rural, a substantial proportion belongs to tribal communities, and specialist ophthalmic services are limited by distance, transport, and cost [9,10]. Although national programs aim to extend outreach, screening, and referral to underserved areas, gaps in availability and uptake of services continue to be reported in remote districts of the North East [9,10]. Despite this, there are almost no community-level data from Tripura on glaucoma awareness, knowledge, or eye care-seeking behavior. To address this gap, we conducted a population-based survey in rural North Tripura to provide the first community estimates of glaucoma awareness, to assess the depth of knowledge and common misconceptions, to document eye-care practices and perceived barriers to examination, and to identify sociodemographic and health-care predictors of awareness.

## Materials and methods

Study design and setting

This community-based cross-sectional survey was conducted between April 1 and June 30, 2025, in North Tripura, India, under the District Residency Programme. Two administrative blocks were selected at random, and within them, four villages were chosen to provide demographic and geographic diversity. The study area is predominantly rural, has a sizeable tribal population, and has limited access to specialist ophthalmic services [9,10]. All interviews were conducted in participants' homes. Written informed consent was obtained from each participant before data collection. The study received institutional ethics approval from Agartala Government Medical College and Govind Ballabh Pant Hospital. All procedures followed the ethical principles of the Declaration of Helsinki [11] and national guidance on research involving human participants issued by the Indian Council of Medical Research [12]. The full questionnaire and translation details are provided as Supplementary File 1.

Eligibility criteria

Adults aged 30 years or older, residents of the selected village for at least one year, and able to provide informed consent were eligible. Exclusion criteria were cognitive impairment limiting reliable response, active psychiatric illness interfering with the interview, or temporary residence in the village (e.g., seasonal workers or visitors). Only one eligible adult per household was interviewed.

Sample size

The minimum required sample size was calculated as 196, based on an expected awareness proportion of 8-10% from prior rural Indian data [5], 95% confidence, 5% absolute precision, a design effect of 1.5 for cluster sampling, and 10% allowance for nonresponse. To permit subgroup analysis and improve precision, we set an operational target of 420 approached adults distributed approximately evenly across the four study villages.

Sampling strategy

We used multistage cluster sampling. Stage 1: Two blocks in North Tripura were randomly selected. Stage 2: Four villages within those blocks were randomly selected. Within each village, we prepared a current household list and applied systematic sampling using a fixed interval. Households absent at the first visit were revisited at least twice at different times before being classified as nonresponders. When more than one eligible adult lived in a sampled household, the Kish method was used to select the respondent [13]. The village was used as the primary cluster for design-effect considerations and for variance estimation in regression analyses.

Questionnaire, translation, and data collection

Data were collected with a structured, pretested questionnaire adapted from prior Indian community surveys of glaucoma awareness, knowledge, and practice [5]. The instrument captured sociodemographic variables (age, sex, education, tribal background), awareness of glaucoma (four core items), knowledge (six items), practice (eye examination in the past 12 months), and reported sources of information and barriers to examination. The questionnaire was translated into Bengali and Kokborok, independently back-translated by bilingual reviewers, and reconciled by consensus; full English and translated versions are provided in Supplementary File 1. Trained medical residents and local health workers administered face-to-face interviews at participants' homes. Interviewers received standardized training on questionnaire content, consent procedures, and use of simple visual aids (e.g., schematic drawings of the eye and optic nerve) to assist participants with limited literacy. Typical interview duration was 10-15 minutes. The tool was piloted in a sample of 30 participants from a nearby village; wording was refined before the main survey.

Operational Definitions and Scoring

Awareness score: One point was assigned for each correct response to four core awareness items (range 0-4). A score of 3 or 4 was classified as good awareness.

Knowledge score: One point was assigned for each correct response to six knowledge items (range 0-6). Scores of 5-6 were classified as good knowledge, 3-4 as moderate, and 0-2 as poor.

Practice: Defined as self-report of having undergone a formal eye examination by an ophthalmologist or optometrist in the previous 12 months (yes or no).

Barriers to examination: Participants who had not undergone an eye examination in the past year were asked reasons; multiple responses were permitted. Options included no perceived need, distance or transport difficulty, and cost, reflecting known barriers in underserved Indian settings, including the North East [9,10].

Data management and handling of missing data

Questionnaire data were double-entered into MS Excel (Microsoft Corporation, Redmond, Washington, United States), checked for range and internal consistency, and imported into IBM SPSS Statistics for Windows, Version 25 (Released 2017; IBM Corp., Armonk, New York, United States) for analysis. Item-level missingness was reviewed prior to analysis; missingness was under 5% for primary variables, and complete-case analysis was used for the main models. For transparency, we conducted sensitivity analyses using multiple imputation for any variables with >5% missing; results were consistent with complete-case findings (Supplementary File 1).

Statistical analysis

Continuous variables are reported as mean ± standard deviation. Categorical variables are reported as numbers and percentages. Group differences were assessed with the chi-square test for categorical variables and with an independent samples t-test or one-way analysis of variance for continuous variables, as appropriate.

To identify factors associated with awareness of glaucoma, we fitted a binary logistic regression model with awareness (ever heard of glaucoma: yes = 1, no = 0) as the dependent variable. Independent variables specified a priori were age (continuous, years), sex (reference = male), education (reference = illiterate; categories: up to 10th standard; graduate or professional), tribal background (reference = no), and prior ophthalmic consultation in the past year (reference = no). Model building retained all specified covariates; adjusted odds ratios with 95% confidence intervals and two-sided p-values are reported. Multicollinearity was assessed with variance inflation factors (VIFs), and all VIFs were <2. Because sampling was clustered by village, standard errors were reestimated using cluster robust variance estimators at the village level; these cluster-adjusted results are reported. Model fit was examined with Hosmer and Lemeshow goodness-of-fit statistics and inspection of residuals. Statistical significance was defined as p < 0.05.

Confidentiality

Interviews were conducted privately. Personal identifiers were not recorded in the analytic dataset. All results are presented in aggregate.

## Results

Participant characteristics

A total of 420 eligible adults were approached, of whom 368 completed the survey (response rate, 87.6%). The mean (±SD) age of respondents was 47.2 ± 13.3 years. Women comprised 198 of 368 participants (53.8%). Educational attainment was as follows: 87 of 368 (23.6%) were illiterate; 192 of 368 (52.2%) had formal education up to the 10th standard; and 89 of 368 (24.2%) were graduates or working professionals. A total of 111 of 368 participants (30.2%) identified as being from a tribal community. These characteristics are summarized in Table 1.

**Table 1:** Demographic characteristics (N = 368)

Variable	n (%) or mean ± SD
Age (years)	47.2 ± 13.3
Female	198 (53.8)
Illiterate	87 (23.6)
Up to the 10th standard	192 (52.2)
Graduate/professional	89 (24.2)
Tribal background	111 (30.2)

Item-level missingness was negligible for primary variables and did not materially affect denominators reported below; all prevalence estimates use the full analytic sample (n = 368) unless otherwise indicated. Where missingness exceeded 5% for any secondary variable, we performed sensitivity checks using multiple imputation; results were consistent with complete-case analyses.

Awareness

**Figure 1:**
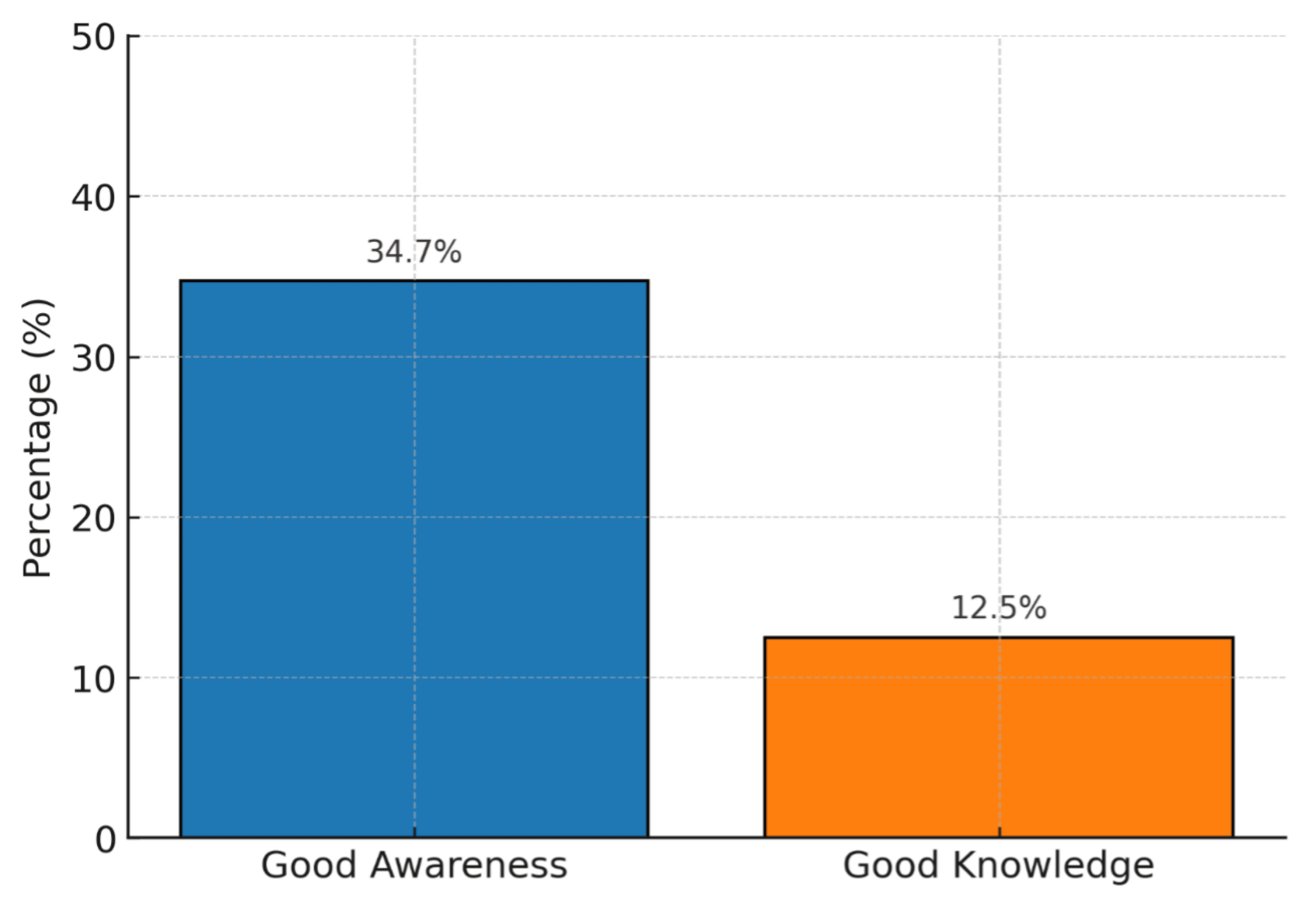
Proportion of participants who had heard of glaucoma (n = 72) achieving good awareness (≥3/4) and good knowledge (5-6/6): 34.7% (25/72) and 12.5% (9/72), respectively

Overall, 72 of 368 participants (19.6%) reported that they had heard of glaucoma. Awareness varied substantially by education. Among those who were illiterate, six of 87 (6.9%) reported awareness; among those educated up to the 10th standard, 28 of 192 (14.6%) reported awareness; and among graduates or professionals, 35 of 89 (39.3%) reported awareness. This gradient was statistically significant (p < 0.001). By sex, awareness was reported by 29 of 170 men (17.1%) and 43 of 198 women (21.7%). Among those who were aware of glaucoma (n = 72), 25 of 72 (34.7%) achieved a “good awareness” score (≥3 out of four items), and nine of 72 (12.5%) achieved “good knowledge” (5-6 out of six items). These proportions are shown in Figure 1 and detailed in Table 2.

**Table 2:** Glaucoma awareness by demographics

Variable	Total n	Aware n (%)
Illiterate	87	6 (6.9)
Up to 10th standard	192	28 (14.6)
Graduate/professional	89	35 (39.3)
Male	170	29 (17.1)
Female	198	43 (21.7)

Knowledge among participants who were aware

Among the 72 participants who had heard of glaucoma, specific knowledge gaps were common. Correct responses for key items were as follows: distinguishing glaucoma from cataract, 33 of 72 (45.8%); recognizing that glaucoma can be asymptomatic in its early course, 18 of 72 (25.0%); acknowledging that a family history may increase risk, 26 of 72 (36.1%); and recognizing that untreated glaucoma can lead to irreversible vision loss, 23 of 72 (31.9%). These findings are summarized in Table 3.

**Table 3:** Knowledge among those aware (n = 72)

Item (correct)	n (%)
Glaucoma ≠ cataract	33 (45.8)
Often asymptomatic early	18 (25.0)
Familial predisposition	26 (36.1)
Irreversible vision loss if untreated	23 (31.9)

Practices and reported barriers to eye examination

In the overall cohort, 39 of 368 participants (10.6%) reported having undergone an eye examination by an ophthalmologist or optometrist within the preceding 12 months. Among those who were aware of glaucoma, 17 of 72 (23.6%) reported such an examination within the past year.

**Figure 2:**
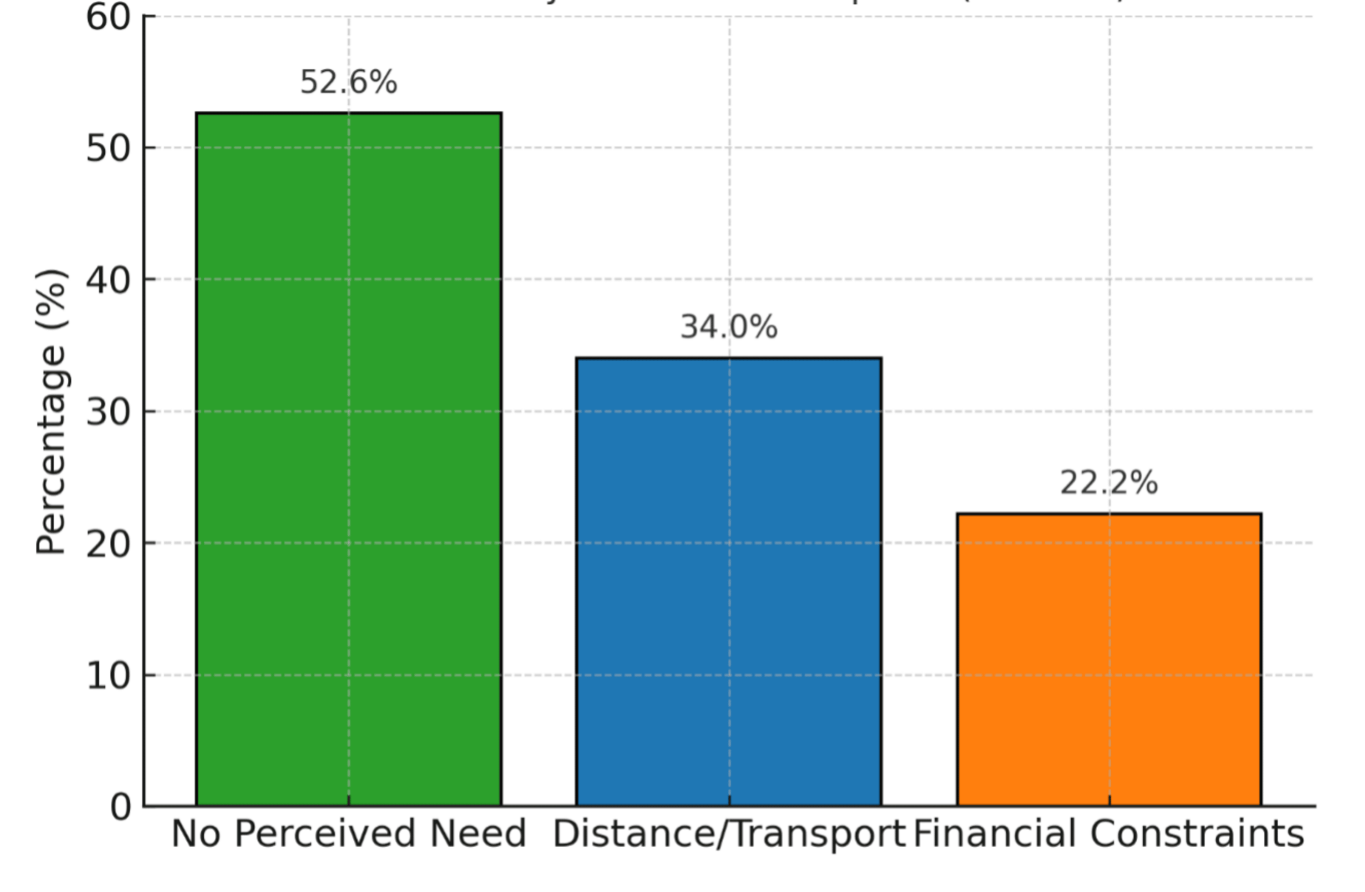
Reported barriers to undergoing eye examinations (N = 368; multiple responses allowed): no perceived need, 52.6% (194/368); distance/transport, 34.0% (125/368); and financial constraints, 22.2% (82/368)

Participants were also asked why they had not undergone an eye examination in the past year; multiple responses were allowed. The most frequently cited barriers were lack of perceived need (“no problem with vision”), reported by 194 of 368 (52.6%); distance or transport limitations, reported by 125 of 368 (34.0%); and financial constraints, reported by 82 of 368 (22.2%). These barriers are illustrated in Figure 2.

Information Sources

**Figure 3:**
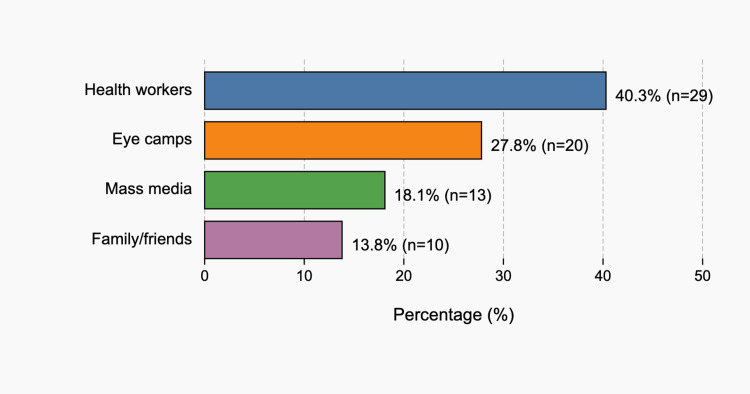
Sources of glaucoma information among participants who had heard of glaucoma (n = 72): health workers, 40.3% (29/72); eye camps, 27.8% (20/72); mass media, 18.1% (13/72); and family/friends, 13.8% (10/72)

Among the 72 participants who had heard of glaucoma, the most common reported sources of information were frontline health workers (29 of 72; 40.3%), eye camps (20 of 72; 27.8%), mass media (13 of 72; 18.1%), and family or friends (10 of 72; 13.8%). These distributions are shown in Figure 3.

Predictors of Awareness

In multivariable logistic regression, higher educational status and prior contact with eye care were independently associated with having heard of glaucoma. Compared with participants with education up to the 10th standard or less, those with graduate or professional education had higher odds of awareness (adjusted odds ratio (AOR), 3.05; 95% confidence interval (CI), 1.87-4.98; p < 0.001). Participants who reported a prior ophthalmic consultation also had higher odds of awareness (AOR, 4.92; 95% CI, 2.68-9.02; p < 0.001). Sex and tribal background were not statistically significant predictors. Full unadjusted and adjusted estimates are shown in Table 4.

**Table 4:** Predictors of glaucoma awareness (binary logistic regression) The adjusted model includes age, sex, education, tribal background, and prior ophthalmic consultation. Standard errors and 95% CIs for adjusted estimates were re-estimated using cluster-robust variance at the village level (four clusters). Variance inflation factors <2 for all covariates. Hosmer-Lemeshow goodness-of-fit p = 0.62, indicating acceptable model fit

Predictor (contrast)	Unadjusted OR (95% CI)	Adjusted OR (AOR) (95% CI)	p-value
Higher education (graduate/professional vs ≤10th)	3.80 (2.11-6.80)	3.05 (1.87-4.98)	<0.001
Prior ophthalmic consultation (yes vs no)	5.60 (3.10-10.1)	4.92 (2.68-9.02)	<0.001
Gender (female vs male)	1.35 (0.80-2.28)	1.22 (0.67-2.23)	0.51
Tribal background (yes vs no)	0.89 (0.50-1.56)	0.82 (0.43-1.57)	0.55
Age (per 1-year increase)	0.98 (0.96-1.00)	0.98 (0.96-1.00)	0.07

Because the study used multistage cluster sampling with village as the primary cluster, standard errors for regression estimates were re-estimated using cluster-robust variance at the village level; cluster-adjusted confidence intervals and p-values are presented in Table 4. VIFs for covariates were all <2, and model diagnostics indicated acceptable fit (Table 4).

Community Interest

When asked whether they would like more information about glaucoma, 189 of 368 respondents (51.4%) said yes. During fieldwork, multiple community leaders informally emphasized the value of recurring village-level eye camps for both education and screening, aligning with the high proportion of participants who identified health workers and eye camps as their main sources of information.

## Discussion

In this community-based survey of adults aged 30 years and older in rural North Tripura, fewer than one in five respondents (19.6%) reported ever having heard of glaucoma. This low level of awareness is consistent with previous population studies from India that have documented poor recognition of glaucoma outside major urban centers [5,8]. The present study extends that evidence to the North Eastern region, where community-level data have been limited [9,10].

Two findings are particularly notable. First, even among those who had heard of glaucoma, understanding was poor. Only about one-third of aware participants showed good awareness, and only one in eight demonstrated good knowledge. Gaps were common in recognizing that glaucoma may not cause early symptoms, that untreated disease leads to irreversible vision loss, and that family history increases risk. Because glaucoma is often asymptomatic in its early stages and vision loss cannot be restored once it occurs [1,3], such misconceptions increase the likelihood of late presentation. Similar patterns have been reported in other Indian surveys where self-reported awareness often reflects name recognition rather than accurate understanding of disease course, risk factors, or the need for regular follow-up [4,8]. These findings highlight a persistent risk of advanced disease at first diagnosis in rural populations.

Second, preventive eye care was uncommon. Only 10.6% of all participants reported an eye examination within the past year, and even among those aware of glaucoma, fewer than one in four had undergone a recent examination. This gap between nominal awareness and actual care seeking mirrors results from other Indian studies showing that most glaucoma cases are still detected at moderate or advanced stages [4,8]. It reinforces that awareness alone is insufficient unless supported by access, affordability, and perceived need.

The reasons participants gave for not seeking eye examinations help explain this disconnect. The most common barriers were no perceived need, distance or transport difficulty, and cost. These same barriers have been described in underserved parts of the North East, where specialist ophthalmic services are distant and outreach camps are often the only contact point with formal eye care [9,10,14]. Similar findings from rural South India show that many adults delay eye checks because they attribute gradual vision loss to aging rather than disease [14]. Together, these patterns emphasize the need to strengthen both awareness and access.

Educational attainment and previous eye care contact emerged as strong predictors of awareness. Participants with graduate or professional education and those reporting prior ophthalmic consultation were far more likely to have heard of glaucoma. These results are consistent with other Indian studies showing that literacy, counseling, and prior exposure to organized eye care are the strongest correlates of glaucoma awareness [5,8,15,16]. In contrast, sex and tribal background were not independent predictors in the adjusted model, suggesting that structural access to services and educational opportunity may be more influential than demographic factors alone.

Economic and logistical barriers remain central. Cost, transport burden, and low perceived urgency are well-recognized causes of underuse of eye care in rural and economically marginalized populations, and they continue to contribute to delayed diagnosis even when people have heard of glaucoma [17,18]. These observations, combined with the present findings, support the need for accessible village-level services and brief, structured counseling that clearly explains that glaucoma can cause permanent vision loss even without pain or early symptoms.

The findings have practical implications for service delivery in North Tripura and similar districts. Respondents who were aware of glaucoma most often cited health workers and eye camps as their source of information, indicating that existing community platforms are the most effective channels for education. Integrating simple, culturally appropriate glaucoma messages into these existing programs could be a pragmatic first step. Such messaging should stress that glaucoma can cause irreversible blindness even in the absence of symptoms, that adults over 40 should have periodic eye checks, and that family history increases risk. Continued support for mobile and subsidized eye care services is also important, given that distance and cost remain major barriers [9,10,14,17,18]. Because prior ophthalmic consultation was strongly associated with awareness, brief counseling during any eye care encounter, for example, at screening camps, refraction visits, or cataract evaluation, may have high yield in increasing understanding and encouraging timely examination.

This study has several strengths. It used probability-based household sampling, a pretested and translated questionnaire administered in local languages, and multivariable analysis to identify independent predictors. The full questionnaire and translations are available as supplementary material to enhance reproducibility. Item-level missingness was minimal, and sensitivity analyses using multiple imputation confirmed the stability of results. Regression standard errors were re-estimated using cluster robust variance to account for the multistage sampling design.

Certain limitations should be acknowledged. The cross-sectional design does not allow causal inference; for instance, it cannot distinguish whether awareness led to consultation or consultation led to awareness. All measures of awareness, knowledge, and prior eye examination were self-reported and are therefore subject to recall and social desirability bias. The questionnaire, although adapted and back-translated, was not formally validated for psychometric reliability across languages, which remains an area for future research. We used cluster robust variance to account for multistage sampling. Because there were four clusters, cluster-based inference may be less precise, and this should be considered when interpreting confidence intervals. Finally, the survey covered four villages in a single district and may not reflect all cultural or health system differences across the North East. Despite these limitations, the patterns observed are consistent with national trends and provide valuable local data to inform program design [9,10].

## Conclusions

Glaucoma awareness and preventive eye-care practices in rural North Tripura remain suboptimal. Despite moderate educational influence, widespread misconceptions and logistical barriers persist. Comparative evidence from other Indian regions underscores enduring rural-urban disparities. Culturally tailored education, mobile eye-care services, and community-integrated screening under the National Programme for Control of Blindness and Visual Impairment (NPCB & VI) framework are essential to enhance early detection and prevent avoidable blindness in North Eastern India.

## Appendices

Supplementary file 1

Questionnaire for Community Survey on Glaucoma Awareness, Knowledge, and Practices

Setting: Rural North Tripura

Target population: Adults aged 30 years and older

Estimated interview time: About 10 to 15 minutes

*Interviewer Instructions*

1. Greet the participant politely and confirm eligibility: age of at least 30 years and resident in the village for at least one year. 2. Read the consent text aloud and obtain written informed consent before proceeding. If the participant cannot sign, mark their fingerprint, and obtain a witness signature, according to local ethics approval. 3. Ask questions exactly as written. If the participant does not understand a term, use the supplied simple explanation note for that item, but do not prompt toward a particular answer. 4. Record responses using the numeric codes provided. If a respondent gives multiple answers where multiple responses are permitted, mark all applicable codes. 5. If the participant declines any question, record refusal with the code 4 and continue unless the Participant wishes to stop the interview. 6. After completion, thank the participant and provide a brief health education leaflet if available.

Consent Text

Hello my name is ________________________ from _______________________. We are conducting a household survey about eye health in this area. Participation is voluntary. If you agree to take part, we will ask you some questions about your background, awareness of eye conditions, and recent use of eye care. The interview will take about 10 to 15 minutes. Your name will not be recorded in the study dataset, and your answers will be kept confidential. You may stop at any time without explanation, and this will not affect any care you receive. Do you agree to participate? 1 Yes, continue with the interview 2 No, thank you, and end the interview

Section A: Participant identification and administrative information

A1 Interview identification number Code __________

A2 Date of interview Date DD MM YYYY __________

A3 Village name Text __________

A4 Household number or unique household id Text __________

A5 Respondent initials Text __________

A6 Interviewer initials Text __________

Section B: Eligibility and screening

B1 Age in completed years Numeric __________

B2 Have you lived in this village for at least one year? 1 Yes 2 No 3 Unsure 4 Refused

If B2 is 2 or 3, end the interview politely and record the reason

Section C: Sociodemographic characteristics

C1 Sex of respondent

1 Male 2 Female 3 Other 4 Refused

C2 What is the highest level of formal education you have completed?

0 No formal schooling 1 Primary completed 2 Up to 10th standard 3 Higher secondary 4 Graduate or professional. 5 Refused

C3 Occupation main activity Text __________ 4 Refused

C4 Do you identify as belonging to a tribal community?

1 Yes 2 No 4 Refused

C5 Household monthly income category

1 Below local poverty threshold 2 Between poverty threshold and median 3 Above median 4 Refused Note for C5 interviewer code using locally defined categories used in the study protocol

Section D: General health and ocular history

D1 Do you have any chronic medical conditions diagnosed by a doctor? Prompt examples: diabetes, hypertension

1 Yes 2 No 4 Refused

If D1 is 1, ask D1a; else skip to D2

D1a Please specify all diagnosed chronic condi%ons separated by comma Text __________ 4 Refused

D2 Have you ever been told by a doctor or health worker that you have poor eyesight or an eye disease?

1 Yes 2 No 4 Refused

D3 In the last 12 months, have you had an eye examination by an ophthalmologist or optometrist?

1 Yes 2 No 4 Refused

If D3 is 1, ask D3a else; skip to Section E

D3a Where was this examination done?

1 Government hospital 2 Private clinic 3 Eye camp 4 Vision centre or primary health centre 5 Other specify __________ 6 Refused

Section E: Awareness of glaucoma

E1 Before today, have you ever heard of something called glaucoma? 1 Yes 2 No 3 Do not know 4 Refused

If E1 is 2 or 3, skip to Section G

If E1 is 1, continue to Section F

Section F: Knowledge about glaucoma

Interviewer note: Explain only if the respondent asks.

Simple explanation: Glaucoma is a condition that can damage the nerve at the back of the eye and can cause loss of vision if not treated. Do not suggest answers.

F1 Can glaucoma be confused with cataract? How is glaucoma different from cataract? Open response, record text, and then code

1 Correct distinction 2 Partially correct 3 Incorrect 4 Refused

Scoring rule for F1 mark 1 for Correct distinction or Partially correct if the respondent mentions optic nerve pressure, painless vision loss, or internal eye pressure; otherwise 0

F2 Is glaucoma usually symptomatic early in the disease, or can it be without symptoms early?

1 Usually has early symptoms 2 Often has no early symptoms 3 Do not know 4 Refused

Correct answer code 2. For scoring, give 1 for a correct response; 2 else 0

F3 Can untreated glaucoma lead to permanent vision loss?

1 Yes 2 No 3 Do not know 4 Refused

Correct answer 1

F4 Can glaucoma run in families, meaning that having a relative with glaucoma increases your chance?

1 Yes 2 No 3 Do not know 4 Refused

Correct answer 1

F5 Have you heard the term high eye pressure or raised intraocular pressure?

1 Yes 2 No 4 Refused

If F5 is 1, ask F5a; else, skip to F6

F5a Do you know that high eye pressure is related to glaucoma?

1 Yes 2 No 3 Do not know 4 Refused

Scoring for F5 and F5a, give 1 if respondent answers yes to F5 and yes to F5a; otherwise 0

F6 If someone has glaucoma, can treatment prevent further vision loss?

1 Yes, treatment can prevent further loss 2 No, treatment cannot help 3 Do not know 4 Refused

Correct answer 1

Knowledge scoring summary

Total knowledge items scored range 0 to 6 as follows: F1, F2, F3, F4, F5/F5a, and F6. For F1, F2, F3, F4, F5/F5a, and F6, assign 1 for each correct response, 0 otherwise. Categorize total knowledge score as 0 to 2, poor; 3 to 4, moderate; 5 to 6, good

Section G Attitudes and perceived need

G1 Do you think adults over 40 should have their eyes checked even if they do not have symptoms?

1 Yes 2 No 3 Do not know 4 Refused

G2 If you noticed a gradual worsening of vision, would you seek professional eye care?

1 Yes, immediately 2 Wait and see 3 Only if vision becomes very poor 4 No 4 Refused

Section H Eye care practices and barriers

H1 Have you ever had surgery for an eye condition?

1 Yes 2 No 4 Refused

H2 If you have not had an eye examination in the past year, what are the reasons? Allow multiple responses; mark all that apply.

1 No perceived need 2 Distance or transport difficulty 3 Cost cannot afford 4 Fear of treatment or surgery 5 Prefer traditional remedies 6 Could not get time from work 7 No one to accompany 8 Other specify __________ 4 Refused

Section I Sources of information

I1 From where did you first hear about glaucoma? If multiple records main source.

1 Frontline health worker or ASHA 2 Eye camp 3 Mass media, radio, TV, newspaper 4 Family or friends 5 Private clinic 6 Other specify __________ 4 Refused

Section J Closing and interest in information

J1 Would you like more information about glaucoma and where to get an eye check?

1 Yes 2 No 4 Refused

J2 If yes, what is the best way to get information at the village level

1 Through the village health worker 2 During eye camps 3 At the primary health centre 4 Local meeting or village notice 5 Printed leaflet 6 Other specify __________ 7 Refused

Interviewer's note at the end

Record any important free-text observations about the respondent's understanding, refusals, or local issues that may affect interpretation

Text __________

Data entry codes and variable names

Provide a variable name list that matches the questionnaire for direct data entry, e.g., E1_awareness E2_F1_distinction, etc. Use numeric codes described above for data entry. Missing values should be coded 4

Pilot testing and translation notes 1. The English questionnaire was piloted in 30 adults in a nearby village not included in the main sample. Pilot results guided minor wording changes for clarity. 2. The questionnaire was translated into Bengali and Kokborok and back-translated independently by bilingual reviewers. Discrepancies were reconciled in a consensus meeting and final versions retained. 3. For transparency, the English source and translated versions are provided as Supplementary File 1. Ethical and data protection notes 1. All interviews were conducted after obtaining written informed consent. 2. Personal identifiers are not collected in the analysis dataset. Unique interview ID links responses to household ID only. 3. Data will be stored on password-protected computers, and only aggregated results will be reported.

Quality control procedures 1. All interviewers received standardized training on the questionnaire, informed consent, and use of visual aids and role-played interviews before fieldwork. 2. Supervisors rechecked 10% of interviews with random visits and cross-checked data entry for a 10% sample. 3. Double data entry was used, and discrepancies reconciled.

Glaucoma saimanmani saimanma tei samungo lukurokni naikolmungni bagwi swngmungni bijap Se9ng Ganao tongnai North Tripura Target borok Borok kotorrok 30 bisini simi Kok salainani jora hwnwi uansukjak ni simi 15 minit jora Kok swngnai borok ni kok 1. Kaham khe hambai rwdi tei wngwi mano hwnwi confirm khlai di: umor 30 bisini swkang tei kamio khoroksa bisini swkang tongnai. 2. Consent text no khorang kotor khe poriwi naidi tei swkang swkang swkang swijak koktwma mankha. Hambainairok sign khaiwi manliya hwnkhe yakni mungwi mark khaidi tei sakhini mungwi sign khaiwi manliya hwnkhe amchaini kerongni swmai tangmani bisingtwi. 3. Swnamjakma hai kheno swngmung swngdi. Par%cipant kaisa term no bujiliya hwnkhe a manwini bagwi rwjak kwchak kuphur khe sajakmani note no use khlai di phiya kaisa answer ni bagwi ta uana di. 4. Number rwjak code no twiwi answer no record khlai di. Kaisa answer rwnai kwbangma answer rwkhe aro kwbangma answer rwnani permission tongo joto applicable code no mark khai di. 5. Jesa phano swngmungno record khwlaiwi rwna bagwi code 4 bai rwngya khe tei interview no rwktharna muchungya khe wngwi tongnai. 6. Phiyaba wngwi thangmani ulo, wngwi thangnai borokno hambai rwdi tei wngwi tongkhe, khoroksa khoroksano hamya wngwi tongmani koktwma leaflet rwdi. Raida swimung Jora ani mung ___________________ ni _______________________. Chwng o jagao mokolni hamkraini bagwi nokni borokrokni survey khlaiwi tongo. Bathakmani abo sakni muchungtwi. Nwng bachana bagwi rwngkhe chwng nono nini background ni mokol ni bemar ni kok no twiwi kiching kiching swngmung swngnai tei mokol no naikolnani kok no twiwi tabuk jorao tamo tamo wngkha. Interview wngna bagwi 10 ni simi 15 minit jora nangnai. Nini mung study dataset o swijak glak tei nini answer no rwjaknai. Nwng jephuru phano explana%on kwrwi khe tongwi mano tei omo nwng mannai jesa phano care no affect khwlaiwi mano. Nwng bachana bagwi rwngkha de? 1 Hoi kok salaimani swkang tongdi 2 Hambai ya tei kok salaimani páithak Sic%on A Bachanairokni sinimung tei samung tangmani koktwma A1 kok salaimani sinijakmani nambar Kod __________ A2 Kok salaimani sal Sal DD MM BISI __________ A3 kami ni mung swijakma __________ A4 nokni mung eba nokni id koktwma __________ A5 Sanaisa swkangni swimung __________ A6 Swnangnai borokni mungni mung __________ Sikson B rwngthoma tei naikolmung B1 Umor páijak bisirokni bisingo __________ B2 Nwng o kamio at least bisi kaisa tongwi tongkha de? 1 Hoi 2 Ya 3 Sichaya 4 Hamya B2 2 eba 3 wngkhe kok salaimungno hamjakma bai pái di tei karonno swi di Sikson C Samajik borokrokni mungwi C1 answer rwnai ni sex 1 chwla 2 bwrwi 3 tei kubun 4 rwjakya C2 Nwng formal swrwngmungni bisingo tamo tamo wngkha? 0 Formal swrwngmung kwrwi 1 Praimari swrwngmung páikha 2 Chisni jora jora 3 Kwchak swrwngmung 4 swrwngmung swrwngmung eba samung khlaina 4 Yakarjak C3 Samung kotor samung Kokthai __________ 4 Yakarjak C4 Nwng sakno tribal dophani borok hwnwi phunuknai? 1 Hoi 2 Ya 4 Hamya C5 Nogo talbrum rang ajimani 1 Amchaini kwrwi kwrwi wngmani threshold ni tola 2 Kwplaisa kwrwi wngmani threshold tei median ni bisingo 3 Median ni sakao 4 Refused C5 kok sanai code ni bagwi note wngkha, porinani protocol o swnamjakmani jagao swnamjaknai Sikson D Kwplaisa sak hamya tei mokolni koktwma D1 Nini thani mungsa swk chronic bemar tongkha de, doctor bai saimanjak? Khakchangma udhahoronrok chini hamya hamya 1 Hoi 2 Ya 4 Yakarya D1 1 wngkhe D1a no swngdi tei D2 no skip khai di. D1a Khapang srangwi bebak saimanjak kwmajak bemarrokno comma bai swkakjak swimung __________ 4 Yakarjak D2 Nwng doctor eba health worker ni thani nini mokol hamya eba mokol ni bemar hwnwi sakha de? 1 Hoi 2 Ya 4 Hamya D3 Last 12 months ni bisingo nwng mokol ni doctor eba mokol ni doctor bai mokol ni exam khaijak kha de? 1 Hoi 2 Ya 4 Hamya D3 1 wngkhe D3a no swngdi tei E o thangdi D3a O exam tamo wngkha? 1 Sorkarni daktal 2 Sakni daktal 3 Mokolni camp 4 Nugwi tongmani jaga eba puila sak hamya wngmani jaga 5 Buini kok sanani __________ 4 Yakarjakya E bwkhak glaucoma ni kokno saimanmani E1 Tini swkang nwng glaucoma hwnwi mungsa swk kok khnakha de? 1 Hai 2 Ya 3 Siya 4 Yakha E1 2 eba 3 wngkhe G ni thani thangdi E1 1 wngkhe F ni thani thangdi. Sec%on F Glaucoma ni kok saimanma Swngnai borokni note Samung rwnai borok swngkhe simi se buj thumdi. Kwplaisa khe sajakmani kok: Glaucoma wngkha mokolni bwskango tongnai nerve no swkakwi mano tei hamya wngwi tongkhe mokol nukya wngwi mano. Jobab suggest khai di. F1 Glaucoma no mokol ni rangchak bai kwthalaiwi mano de? Glaucoma bai mokolni wngwi bahai khe kwrwng? Phiyaba jawab record swijak tei aboni ulo code 1 Kaham khe swlaijak 2 Kisa khe kaham 3 Chaya 4 Yakarjak F1 ni bagwi scoring rule mark 1 ni bagwi Kaham khe swlaijakmani eba Kaham khe swlaijakmani, jawab rwnai borok mokolni nerve ni chapmung hamya kwrwi nukmung kwmajak eba mokolni bisingo chapmung wngmani kok sakhe; abohai wngyakhe 0 F2 Glaucoma wngmani jorao swkangni simino nukjaknai eba swkangni jorao nukjakya wngwi mano de? 1 Kwbangma jorao swkangni mari nukjago 2 Kwbangma jorao swkangni mari nukya 3 Siya 4 Refused Kwthar answer code 2. Scoring ni bagwi 1 rwdi thik answer ni bagwi 2 else 0 F3 Glaucoma no hamjakya khe mokol nukya wngwi mano de? 1 Hai 2 Ya 3 Siya 4 Yakha Kwthar jobab 1 F4 Mokolni bemar nukhungrogo tongwi mano de, aboni majra wngkha mokolni bemar bai tongnai borok tongmani inc
